# Supplementary material for: Unhealthy food consumption among 20–59 years old adults in Bangladesh: Findings from a nationally representative cross-sectional survey
Source: PLoS One. 2025 Dec 2;20(12):e0336984. doi: 10.1371/journal.pone.0336984 (PMC12671833; doi:10.1371/journal.pone.0336984)
Supplement: S1 Table — (DOCX) [file pone.0336984.s001.docx]

S1 Table. Prevalence of SFS, SS and SSBs consumption (last 7 days) by geographical and socio-demographic stratum of the study participants

| **Variables** | **Men** | | | | | | **Women** | | | | | |
| --- | --- | --- | --- | --- | --- | --- | --- | --- | --- | --- | --- | --- |
|  | **SFS** | | **SS** | | **SSBs** | | **SFS** | | **SS** | | **SSBs** | |
|  | **%** | **95% CI** | **%** | **95% CI** | **%** | **95% CI** | **%** | **95% CI** | **%** | **95% CI** | **%** | **95% CI** |
| **Overall** | 57.8 | 52.4, 62.9 | 75.2 | 69.3, 80.3 | 77.3 | 72.0, 81.9 | 29.6 | 24.7, 34.9 | 58.7 | 51.5, 65.5 | 35.3 | 26.0, 45.8 |
| **Age in years** |  | | | | | | | | | | | |
| 20-29 | 69.3 | 63.4, 74.6 | 80.6 | 74.9, 85.3 | 77.3 | 70.9, 82.6 | 35.4 | 29.5, 41.8 | 66.3 | 58.5, 73.4 | 38.2 | 28.4, 49.1 |
| 30-39 | 59.1 | 52.9, 65.0 | 77.1 | 71.2, 82.1 | 77.6 | 71.0, 83.0 | 31.4 | 26.4, 36.8 | 56.6 | 48.2, 64.8 | 34.2 | 24.1, 46.0 |
| 40-49 | 53.0 | 45.4, 60.6 | 71.9 | 63.1, 79.3 | 80.5 | 74.6, 85.3 | 25.6 | 19.8, 32.5 | 55.1 | 46.8, 63.2 | 35.1 | 25.3, 46.4 |
| 50-59 | 44.0 | 36.7, 51.5 | 68 | 60.0, 75.1 | 73 | 65.2, 79.6 | 18.5 | 13.3, 25.0 | 51.6 | 44.4, 58.8 | 31.3 | 22.7, 41.5 |
| **Division** |  | | | | | | | | | | | |
| Dhaka | 52.3 | 45.9, 58.7 | 77.7 | 71.6, 82.8 | 78.2 | 62.6, 88.5 | 27.2 | 21.1, 34.4 | 61.3 | 52.4, 69.5 | 35.9 | 24.6, 49.0 |
| Chattogram | 82.3 | 77.1, 86.5 | 87.5 | 77.7, 93.4 | 95.2 | 92.2, 97.1 | 49.7 | 43.9, 55.5 | 83.2 | 70.9, 91.0 | 89 | 83.3, 92.9 |
| Rajshahi | 42.3 | 34.8, 50.1 | 57.2 | 44.6, 68.9 | 63.3 | 54.6, 71.3 | 16.6 | 10.5, 25.3 | 37.9 | 26.3, 50.9 | 16.3 | 9.6, 26.4 |
| Khulna | 65.3 | 60.7, 69.7 | 85 | 81.1, 88.3 | 68.8 | 59.6, 76.6 | 34.9 | 28.1, 42.3 | 70.8 | 66.1, 75.1 | 7.3 | 5.3, 10.1 |
| Barisal | 62.1 | 53.2, 70.3 | 85.2 | 75.0, 91.7 | 92.2 | 85.9, 95.8 | 48.5 | 35.7, 61.5 | 69.3 | 52.7, 82.1 | 52.1 | 36.9, 67.0 |
| Sylhet | 56.8 | 49.5, 63.7 | 89.8 | 80.1, 95.1 | 90.9 | 85.4, 94.5 | 24.6 | 18.4, 32.2 | 77.9 | 74.6, 80.8 | 85.8 | 80.5, 89.8 |
| Rangpur | 57.2 | 51.0, 63.3 | 70.7 | 63.0, 77.4 | 78.6 | 70.6, 85.0 | 21 | 14.0, 30.2 | 46.8 | 43.8, 49.8 | 34.6 | 18.2, 55.8 |
| Mymensingh | 43.3 | 37.6, 49.2 | 74.5 | 70.2, 78.3 | 84.9 | 79.1, 89.4 | 24.1 | 19.2, 29.7 | 49.6 | 38.6, 60.7 | 18.3 | 13.4, 24.4 |
| **Place of residence** |  | | | | | | | | | | | |
| Rural | 57.2 | 51.7, 62.6 | 75.2 | 69.0, 80.5 | 76.7 | 71.2, 81.5 | 28.9 | 24.0, 34.4 | 58.5 | 51.0, 65.6 | 33.8 | 24.3, 44.7 |
| Non-slum urban | 69.0 | 59.9, 76.8 | 73.8 | 64.7, 81.3 | 90.7 | 89.2, 91.9 | 44 | 34.7, 53.8 | 63.1 | 55.1, 70.4 | 71.8 | 60.4, 80.9 |
| Slum | 66.0 | 54.1, 76.2 | 81.9 | 75.1, 87.1 | 87.1 | 82.0, 90.9 | 48.4 | 39.9, 57.0 | 62.8 | 53.3, 71.3 | 61.6 | 41.5, 78.3 |
| **Religion** |  | | | | | | | | | | | |
| Islam | 56.3 | 50.8, 61.7 | 74.5 | 68.1, 80.0 | 75.8 | 70.2, 80.8 | 29.2 | 24.4, 34.6 | 57.4 | 50.2, 64.4 | 32.8 | 23.9, 43.1 |
| Others^a^ | 65.4 | 55.8, 73.8 | 78.9 | 67.1, 87.3 | 85.3 | 77.2, 90.8 | 31.6 | 21.3, 44.2 | 66.4 | 51.5, 78.6 | 50.7 | 30.2, 70.9 |
| **Marital status** |  | | | | | | | | | | | |
| Currently married | 55.4 | 49.6, 61.2 | 73.5 | 67.2, 79.1 | 77.7 | 72.5, 82.3 | 30.4 | 25.2, 36.1 | 59.2 | 51.7, 66.4 | 34.9 | 25.6, 45.5 |
| Others^b^ | 68.9 | 61.1, 75.8 | 83.2 | 77.1, 87.9 | 75.4 | 66.6, 82.5 | 21.5 | 16.8, 27.1 | 53.8 | 44.2, 63.1 | 38.8 | 27.6, 51.3 |
| **Education** |  | | | | | | | | | | | |
| No formal education | 42.9 | 36.8, 49.3 | 65.9 | 58.0, 72.9 | 72.2 | 64.7, 78.6 | 20.7 | 16.3, 26.0 | 48.9 | 41.7, 56.2 | 26.3 | 18.4, 36.2 |
| Primary (grade 1-5) | 62.1 | 55.3, 68.4 | 78.1 | 72.4, 83.0 | 78.5 | 69.8, 85.2 | 28.2 | 23.8, 33.1 | 55.1 | 48.1, 62.0 | 30.9 | 22.8, 40.4 |
| Secondary (grade 6-10) | 64.4 | 58.7, 69.8 | 77.6 | 71.3, 82.9 | 80.3 | 74.2, 85.2 | 35 | 28.3, 42.2 | 66.7 | 58.3, 74.2 | 44.3 | 32.4, 56.9 |
| Higher Secondary & above | 67.6 | 61.1, 73.4 | 84.1 | 78.6, 88.4 | 80.4 | 73.8, 85.6 | 46.1 | 35.2, 57.3 | 75.7 | 65.9, 83.4 | 47.2 | 35.0, 59.7 |
| **Occupation** |  | | | | | | | | | | | |
| Not working | 66.1 | 58.0, 73.4 | 77.2 | 68.3, 84.2 | 80.4 | 75.2, 84.7 | 29.7 | 24.7, 35.3 | 59.1 | 52.0, 65.9 | 35.4 | 26.1, 46.0 |
| Working | 57.0 | 51.4, 62.5 | 75 | 69.1, 80.1 | 77.1 | 71.5, 81.8 | 27.2 | 22.3, 32.7 | 53 | 38.5, 67.0 | 33.3 | 21.0, 48.4 |
| **Wealth quintile** |  | | | | | | | | | | | |
| Poorest | 49.1 | 40.0, 58.2 | 67.4 | 59.0, 74.9 | 71.3 | 62.3, 78.9 | 24.4 | 18.8, 30.9 | 50.4 | 42.6, 58.2 | 27.5 | 20.1, 36.5 |
| Poorer | 58.1 | 51.1, 64.7 | 77 | 69.5, 83.0 | 82.5 | 76.8, 87.0 | 29.1 | 23.1, 36.0 | 57.4 | 48.1, 66.2 | 29.7 | 19.9, 41.9 |
| Middle | 62.3 | 55.6, 68.5 | 75.5 | 68.5, 81.4 | 76.9 | 69.3, 83.1 | 28 | 22.9, 33.9 | 62.4 | 54.2, 70.0 | 34.5 | 23.7, 47.2 |
| Richer | 59.3 | 53.5, 64.9 | 77.4 | 70.8, 82.9 | 76.5 | 69.9, 82.0 | 28.1 | 22.1, 35.1 | 57.2 | 48.4, 65.5 | 40.1 | 29.5, 51.7 |
| Richest | 62.7 | 56.1, 68.8 | 81.1 | 74.4, 86.4 | 79.3 | 71.6, 85.3 | 43.5 | 34.7, 52.7 | 71.3 | 63.5, 78.1 | 53.1 | 40.0, 65.8 |
| **Physical Activity** |  |  | | | | | | | | | | |
| >=150 Minutes/week | 57.8 | 52.4, 63.0 | 76.5 | 71.3, 81.1 | 76.9 | 71.7, 81.5 | 28.9 | 24.1, 34.2 | 58.5 | 51.1, 65.5 | 34.1 | 24.8, 44.9 |
| <150 Minutes/week | 57.7 | 48.3, 66.6 | 69.9 | 61.3, 77.4 | 78.9 | 69.8, 85.8 | 36.3 | 27.8, 45.7 | 60.7 | 50.3, 70.3 | 47.2 | 33.5, 61.4 |
| **Fruits and vegetables intake** |  | | | | | | | | | | | |
| >= 5 servings/day | 61.3 | 54.6, 67.6 | 75.2 | 70.8, 79.2 | 79.5 | 71.4, 85.7 | 41.4 | 32.9, 50.4 | 66.6 | 58.0, 74.2 | 34.0 | 24.4, 45.1 |
| <5 servings/day | 56.9 | 51.1, 62.6 | 75.2 | 68.2, 81.1 | 76.8 | 70.9, 81.8 | 28.3 | 23.6, 33.6 | 57.9 | 50.4, 65.0 | 35.4 | 25.9, 46.2 |
| **Sedentary time** |  | | | | | | | | | | | |
| <= 7 hours | 56.1 | 50.4, 61.7 | 75.2 | 68.8, 80.6 | 76.3 | 70.6, 81.1 | 29.2 | 24.3, 34.8 | 57.5 | 49.9, 64.8 | 34.4 | 24.8, 45.5 |
| >7hours | 64.3 | 58.2, 69.9 | 75.2 | 69.3, 80.3 | 81.6 | 74.7, 86.9 | 31 | 24.1, 38.9 | 64.2 | 54.7, 72.7 | 39.2 | 28.1, 51.6 |
| **Duration of watching TV** |  | | | | | | | | | | | |
| <=4 hours | 57.5 | 52.1, 62.8 | 75.6 | 69.5, 80.8 | 77.1 | 71.5, 81.8 | 29.1 | 24.1, 34.5 | 58.8 | 51.4, 65.8 | 34.4 | 25.0, 45.1 |
| >4hours | 64.5 | 54.3, 73.6 | 64.5 | 57.0, 71.3 | 85.4 | 74.1, 92.3 | 44.7 | 33.7, 56.2 | 56.5 | 48.4, 64.2 | 62.9 | 44.3, 78.2 |
| **Current smoker** |  | | | | | | | | | | | |
| No | 56.3 | 51.3, 61.1 | 72.6 | 65.4, 78.8 | 69.9 | 63.2, 75.7 | 29.5 | 24.7, 34.9 | 58.7 | 51.5, 65.5 | 35.2 | 26.0, 45.7 |
| Yes | 59.9 | 53.2, 66.2 | 78.8 | 73.1, 83.6 | 87.8 | 83.6, 91.0 | 35.2 | 21.2, 52.5 | 56.9 | 37.9, 74.1 | 62.8 | 39.3, 81.5 |
| **Body mass Index (BMI)** |  | | | | | | | | | | | |
| Underweight | 50.6 | 41.9, 59.2 | 74.4 | 66.5, 81.1 | 77.1 | 67.8, 84.3 | 27.8 | 21.4, 35.4 | 53.1 | 43.9, 62.1 | 29.5 | 19.9, 41.5 |
| Normal | 57.4 | 52.2, 62.4 | 74.5 | 67.8, 80.1 | 76 | 69.8, 81.3 | 27.8 | 22.3, 34.0 | 55 | 46.9, 62.9 | 31.3 | 22.2, 42.0 |
| Overweight and/or obese | 61.7 | 54.9, 68.0 | 76.6 | 71.1, 81.4 | 79.4 | 73.6, 84.3 | 31.5 | 26.2, 37.3 | 63.1 | 55.3, 70.3 | 39.9 | 29.9, 50.7 |
| **Hypertension** |  | | | | | | | | | | | |
| Non-hypertensive | 58.5 | 53.0, 63.8 | 74.7 | 68.3, 80.1 | 77.3 | 71.8, 82.0 | 30 | 25.2, 35.2 | 57.4 | 49.9, 64.7 | 33.7 | 24.6, 44.2 |
| Hypertensive | 54.5 | 48.1, 60.9 | 77.3 | 70.4, 83.0 | 77.6 | 69.9, 83.7 | 28.3 | 22.2, 35.3 | 62.6 | 54.3, 70.2 | 40.2 | 29.9, 51.4 |
| **Self -reported heart disease** |  | | | | | | | | | | | |
| No | 58.3 | 53.0, 63.4 | 75.3 | 69.2, 80.4 | 77.4 | 72.1, 82.0 | 29.6 | 24.6, 35.2 | 58.9 | 51.7, 65.7 | 35.2 | 26.0, 45.7 |
| Yes | 50.6 | 41.7, 59.4 | 74.2 | 66.7, 80.5 | 76 | 66.8, 83.2 | 28.9 | 22.2, 36.7 | 57.2 | 47.0, 66.8 | 36.2 | 23.7, 50.9 |
| **Self- reported asthma** |  | | | | | | | | | | | |
| No | 58.3 | 53.0, 63.5 | 75.2 | 69.2, 80.3 | 77.3 | 71.7, 82.0 | 29.1 | 24.2, 34.5 | 59.0 | 51.6, 66.1 | 35.0 | 25.8, 45.5 |
| Yes | 48.5 | 37.8, 59.3 | 75.3 | 64.0, 83.9 | 78.7 | 68.7, 86.2 | 36.2 | 28.6, 44.6 | 53.9 | 45.4, 62.1 | 38.7 | 27.0, 51.9 |
| **Self- reported diabetes** |  | | | | | | | | | | | |
| No | 57.9 | 52.5, 63.1 | 75.7 | 69.9, 80.7 | 78.0 | 72.6, 82.6 | 29.7 | 24.8, 35.1 | 58.7 | 51.6, 65.4 | 35.0 | 25.7, 45.5 |
| Yes | 54.9 | 44.1, 65.3 | 59.8 | 44.8, 73.2 | 57.2 | 44.9, 68.6 | 26.7 | 18.3, 37.1 | 59.1 | 48.4, 68.9 | 40.1 | 29.2, 51.9 |

^a^Hindu, Christian, Buddhist together

^b^Never married, separated, divorced, widowed
