# Supplementary material for: Unhealthy food consumption among 20–59 years old adults in Bangladesh: Findings from a nationally representative cross-sectional survey
Source: PLoS One. 2025 Dec 2;20(12):e0336984. doi: 10.1371/journal.pone.0336984 (PMC12671833; doi:10.1371/journal.pone.0336984)
Supplement: S2 Table — (DOCX) [file pone.0336984.s002.docx]

**S2 Table. Multicollinearity and goodness-of-fit statistics for SFS consumption among men and women**

| **Variables** | **Men** | **Women** |
| --- | --- | --- |
|  | **Variance inflation factor (VIF)** | **Variance inflation factor (VIF)** |
| Duration of watching TV | 1.84 | 2.03 |
| Physical activity | 1.43 | 1.84 |
| Marital status | 1.4 | 1.05 |
| Occupation | 1.39 | 1.06 |
| Sedentary time | 1.39 | 1.42 |
| Education | 1.37 | 1.14 |
| Fruits and vegetables intake | 1.32 | 1.02 |
| Body mass index (BMI) | 1.2 | 1.11 |
| Wealth quintile | 1.15 | 1.23 |
| Place of residence | 1.14 | 1.16 |
| Hypertension | 1.12 | 1.05 |
| Current smoking | 1.1 |  |
| Division | 1.08 | 1.1 |
| Self- reported diabetes | 1.06 |  |
| Self -reported heart disease | 1.06 |  |
| Self- reported asthma | 1.03 |  |
| **Mean VIF** | 1.26 | 1.27 |
| **Goodness of fit of Poisson regression model** |  |  |
| Deviance goodness-of-fit | 2823.665* | 3444.803* |
| Pearson goodness-of-fit | 1935.86* | 3317.694* |

*Corresponding p value=1
