# Supplementary material for: Unhealthy food consumption among 20–59 years old adults in Bangladesh: Findings from a nationally representative cross-sectional survey
Source: PLoS One. 2025 Dec 2;20(12):e0336984. doi: 10.1371/journal.pone.0336984 (PMC12671833; doi:10.1371/journal.pone.0336984)
Supplement: S4 Table — (DOCX) [file pone.0336984.s004.docx]

**S4 Table. Multicollinearity and goodness-of-fit statistics for SSBs consumption among men and women**

| **Variables** | **Men** | **Women** |
| --- | --- | --- |
|  | **Variance inflation factor (VIF)** | **Variance inflation factor (VIF)** |
| Duration of watching TV | 1.5 | 2.03 |
| Physical activity | 1.41 | 1.84 |
| Marital status |  | 1.05 |
| Religion |  | 1.02 |
| Occupation |  | 1.06 |
| Sedentary time | 1.38 | 1.43 |
| Education | 1.19 |  |
| Fruits and vegetables intake |  | 1.02 |
| Wealth quintile | 1.14 | 1.23 |
| Place of residence | 1.13 | 1.16 |
| Hypertension |  | 1.06 |
| Current smoking | 1.06 |  |
| Division | 1.05 |  |
| Self- reported diabetes | 1.02 |  |
| Self- reported asthma |  | 1.01 |
| **Mean VIF** | 1.21 | 1.23 |
| **Goodness of fit of Poisson regression model** | | |
| Deviance goodness-of-fit | 1359.625* | 2733.059* |
| Pearson goodness-of-fit | 778.7229* | 2392.854* |

*Corresponding p value=1
