# Supplementary material for: Unhealthy food consumption among 20–59 years old adults in Bangladesh: Findings from a nationally representative cross-sectional survey
Source: PLoS One. 2025 Dec 2;20(12):e0336984. doi: 10.1371/journal.pone.0336984 (PMC12671833; doi:10.1371/journal.pone.0336984)
Supplement: S5 Table — (DOCX) [file pone.0336984.s005.docx]

S5 Table. Crude prevalence ratios (CPR) and adjusted prevalence ratios (APR) of the factors of SFS consumption among men and women

| **Variables** | **Men** | | **Women** | |
| --- | --- | --- | --- | --- |
|  | **CPR (95% CI)** | **APR (95% CI)** | **CPR (95% CI)** | **APR (95% CI)** |
| **Age in years** |  | | | |
| 20-29 | 1.53 (1.42, 1.66) *** | 1.39 (1.28, 1.52) *** | 1.74 (1.49, 2.03) *** | 1.36 (1.15, 1.61) *** |
| 30-39 | 1.33 (1.23,1.44) *** | 1.23 (1.13, 1.33) *** | 1.52 (1.30, 1.77) *** | 1.25 (1.07, 1.47) ** |
| 40-49 | 1.19 (1.10,1.30) *** | 1.15 (1.06, 1.25) ** | 1.26 (1.06, 1.49) ** | 1.15 (0.98, 1.37) |
| 50-59 | Ref | Ref | Ref | Ref |
| **Division** |  | | | |
| Dhaka | 0.78 (0.71, 0.86) *** | 0.77 (0.70, 0.85) *** | 0.87 (0.75, 1.00) | 0.81 (0.69, 0.94) ** |
| Chattogram | 1.14 (1.07, 1.22) *** | 1.12 (1.05, 1.20) ** | 1.20 (1.0  6, 1.36) ** | 1.16 (1.02, 1.31) * |
| Rajshahi | 0.86 (0.79, 0.93) *** | 0.88 (0.81, 0.96) ** | 0.82 (0.71, 0.95) ** | 0.85 (0.73, 0.98) * |
| Khulna | Ref | Ref | Ref | Ref |
| Barisal | 0.98 (0.90, 1.06) | 0.98 (0.90, 1.06) | 1.26 (1.10, 1.43) ** | 1.27 (1.11, 1.46) ** |
| Sylhet | 0.86 (0.78, 0.95) ** | 0.88 (0.79, 0.96) ** | 0.78 (0.66, 0.93) ** | 0.82 (0.69, 0.98) * |
| Rangpur | 0.83 (0.76, 0.90) *** | 0.86 (0.79, 0.94) ** | 0.60 (0.51, 0.71) *** | 0.63 (0.53, 0.74) *** |
| Mymensingh | 0.69 (0.63, 0.76) *** | 0.68 (0.62, 0.75) *** | 0.56 (0.48, 0.67) *** | 0.57 (0.48, 0.68) *** |
| **Place of residence** |  | | | |
| Rural | Ref | Ref | Ref | Ref |
| Non-slum urban | 1.13 (1.07, 1.20) *** | 1.11 (1.03, 1.19) ** | 1.43 (1.31, 1.56) *** | 1.19 (1.06, 1.35) ** |
| Slum | 1.13 (1.06, 1.21) *** | 1.12 (1.05, 1.20) ** | 1.25 (1.12, 1.40) *** | 1.18 (1.05, 1.33) ** |
| **Religion** |  | | | |
| Islam | Ref | Ref | Ref | Ref |
| Others^a^ | 1.02 (0.95, 1.08) | N/A | 1.03 (0.92, 1.15) | N/A |
| **Marital status** |  | | | |
| Currently married | Ref | Ref | Ref | Ref |
| Others^b^ | 1.22 (1.16, 1.29) *** | 1.03 (0.96, 1.10) | 0.84(0.72, 0.98) * | 0.88 (0.76, 1.02) |
| **Education** |  | | | |
| No formal education | Ref | Ref | Ref | Ref |
| Primary (grade 1-5) | 1.26 (1.18, 1.34) *** | 1.14 (1.07, 1.22) *** | 1.36 (1.21, 1.53) *** | 1.20 (1.07, 1.35) ** |
| Secondary (grade 6-10) | 1.35 (1.27, 1.45) *** | 1.22 (1.14, 1.30) *** | 1.67 (1.50, 1.85) *** | 1.38 (1.23, 1.56) *** |
| Higher secondary & above | 1.38 (1.29, 1.49) *** | 1.28 (1.17, 1.39) *** | 2.04 (1.79, 2.32) *** | 1.63 (1.41, 1.89) *** |
| **Occupation** |  | | | |
| Not working/homemaker | Ref | Ref | Ref | Ref |
| Working | 0.9 (0.83, 0.97) ** | 1.07 (0.97, 1.17) | 1.13 (1.01, 1.27) * | 1.12 (1.00, 1.26) |
| **Wealth quintile** |  | | | |
| Poorest | Ref | Ref | Ref | Ref |
| Poorer | 1.11 (1.03, 1.20) ** | 1.12 (1.04, 1.20) ** | 1.16 (1.01, 1.33) * | 1.15 (1.01, 1.32) * |
| Middle | 1.14 (1.06, 1.23) ** | 1.11 (1.04, 1.20) ** | 1.22 (1.06, 1.40) ** | 1.15 (1.01, 1.32) * |
| Richer | 1.14 (1.05, 1.23) ** | 1.08 (1.00, 1.17) * | 1.38 (1.21, 1.56) *** | 1.19 (1.04, 1.36) * |
| Richest | 1.21 (1.12, 1.30) *** | 1.10 (1.02, 1.19) * | 1.69 (1.50, 1.91) *** | 1.33 (1.16, 1.53) *** |
| **Physical activity** |  | | | |
| >=150 Minutes/week | Ref | Ref | Ref | Ref |
| <150 Minutes/week | 1.06 (1.01,1.12) * | 0.96 (0.90, 1.02) | 1.37 (1.25, 1.51) *** | 1.11 (0.97, 1.26) |
| **Fruits and vegetables intake** |  | | | |
| >= 5 servings/day | Ref | Ref | Ref | Ref |
| <5 servings/day | 0.96 (0.91,1.01) | 0.94 (0.88, 1.00) * | 0.89 (0.79, 0.99) * | 0.81 (0.72, 0.91) *** |
| **Sedentary time** |  | | | |
| <= 7 hours | Ref | Ref | Ref | Ref |
| >7hours | 1.08 (1.03,1.14) ** | 1.01 (0.96, 1.08) | 1.17 (1.07, 1.28) *** | 1.00 (0.90, 1.11) |
| **Duration of watching TV** |  | | | |
| <=4 hours | Ref |  | Ref |  |
| >4hours | 1.16 (1.08, 1.25) *** | 0.93 (0.83, 1.03) | 1.32 (1.17, 1.48) *** | 0.85 (0.71, 1.03) |
| **Current smoker** |  |  |  |  |
| No | Ref | Ref | Ref | Ref |
| Yes | 1.04 (1.00, 1.09) | 1.10 (1.05, 1.15) *** | 0.80 (0.49, 1.30) | N/A |
| **Body mass Index (BMI)** |  | | | |
| Underweight | Ref | Ref | Ref | Ref |
| Normal | 1.02 (0.96, 1.10) | 1.02 (0.95, 1.09) | 1.07 (0.92, 1.24) | 0.95 (0.82, 1.10) |
| Overweight and/or obese | 1.09 (1.02, 1.17) * | 1.06 (0.98, 1.14) | 1.24 (1.07, 1.43) ** | 0.98 (0.85, 1.13) |
| **Hypertension** |  | | | |
| Non-hypertensive | Ref | Ref | Ref | Ref |
| Hypertensive | 0.93 (0.87, 0.98) * | 0.97 (0.91, 1.03) | 0.98 (0.89, 1.07) | 1.03 (0.94, 1.13) |
| **Self -reported heart disease** |  | | | |
| No | Ref | Ref | Ref | Ref |
| Yes | 0.86 (0.77, 0.96) ** | 0.95 (0.86, 1.06) | 0.96 (0.83, 1.10) | N/A |
| **Self- reported asthma** |  | | | |
| No | Ref | Ref | Ref | Ref |
| Yes | 0.92 (0.83, 1.03) | 0.99 (0.89, 1.10) | 1.07 (0.92, 1.24) | N/A |
| **Self- reported diabetes** |  | | | |
| No | Ref | Ref | Ref | Ref |
| Yes | 0.85 (0.74,0.98) * | 0.92 (0.8, 1.06) | 0.93 (0.78, 1.10) | N/A |

*p<0.05; **p<0.01; ***p<0.001

^a^Hindu, Christian, Buddhist together

^b^Never married, separated, divorced, widowed
