# Supplementary material for: Unhealthy food consumption among 20–59 years old adults in Bangladesh: Findings from a nationally representative cross-sectional survey
Source: PLoS One. 2025 Dec 2;20(12):e0336984. doi: 10.1371/journal.pone.0336984 (PMC12671833; doi:10.1371/journal.pone.0336984)
Supplement: S7 Table — (DOCX) [file pone.0336984.s007.docx]

S7 Table. Crude prevalence ratios (CPR) and adjusted prevalence ratios (APR) of the factors of SSBs consumption among men and women

| **Variables** | **Men** | | **Women** | |
| --- | --- | --- | --- | --- |
|  | **CPR (95% CI)** | **APR (95% CI)** | **CPR (95% CI)** | **APR (95% CI)** |
| **Age in years** |  | | | |
| 20-29 | 1.01 (0.97,1.05) | 0.97 (0.93, 1.01) | 1.15 (1.04, 1.28) ** | 0.92 (0.83, 1.03) |
| 30-39 | 1.04 (1.01, 1.08) * | 1.01 (0.98, 1.05) | 1.18 (1.06, 1.31) ** | 1.00 (0.91, 1.11) |
| 40-49 | 1.05 (1.01, 1.09) * | 1.03 (0.99, 1.07) | 1.15 (1.03, 1.28) * | 1.06 (0.96, 1.18) |
| 50-59 | Ref | Ref | Ref | Ref |
| **Division** |  | | | |
| Dhaka | 1.08 (1.02, 1.14) * | 1.06 (1.00, 1.13) * | 2.30 (1.95, 2.71) *** | 1.97 (1.69, 2.29) *** |
| Chattogram | 1.22 (1.16, 1.28) *** | 1.21 (1.15, 1.26) *** | 3.78 (3.26, 4.39) *** | 3.59 (3.13, 4.12) *** |
| Rajshahi | 0.98 (0.92, 1.04) | 0.98 (0.92, 1.04) | 1.56 (1.30, 1.86) *** | 1.60 (1.37, 1.88) *** |
| Khulna | Ref | Ref | Ref | Ref |
| Barisal | 1.22 (1.16,1.29) *** | 1.22 (1.16, 1.29) *** | 2.73 (2.32, 3.22) *** | 2.83 (2.44, 3.29) *** |
| Sylhet | 1.23 (1.17,1.29) *** | 1.22 (1.16, 1.29) *** | 4.12 (3.55, 4.78) *** | 4.50 (3.91, 5.17) *** |
| Rangpur | 1.14 (1.08, 1.2) *** | 1.16 (1.10, 1.22) *** | 1.80 (1.52, 2.14) *** | 1.95 (1.67, 2.28) *** |
| Mymensingh | 1.14 (1.08, 1.2) *** | 1.13 (1.07, 1.19) *** | 1.34 (1.11, 1.61) ** | 1.40 (1.18, 1.66) *** |
| **Place of residence** |  | | | |
| Rural | Ref | Ref | Ref | Ref |
| Non-slum urban | 1.14 (1.11, 1.17) *** | 1.12 (1.08, 1.16) *** | 2.04 (1.92, 2.16) *** | 1.75 (1.62, 1.89) *** |
| Slum | 1.10 (1.07, 1.14) *** | 1.08 (1.05, 1.12) *** | 1.92 (1.80, 2.05) *** | 1.74 (1.61, 1.88) *** |
| **Religion** |  | | | |
| Islam | Ref | Ref | Ref | Ref |
| Others^a^ | 1.01 (0.98, 1.04) | N/A | 1.22 (1.14, 1.31) *** | 1.10 (1.03, 1.17) ** |
| **Marital status** |  | | | |
| Currently married | Ref | Ref | Ref | Ref |
| Others^b^ | 0.98 (0.95, 1.02) | N/A | 1.17 (1.07, 1.28) ** | 1.11 (1.02, 1.21) * |
| **Education** |  | | | |
| No formal education | Ref | Ref | Ref | Ref |
| Primary (grade 1-5) | 1.02 (0.99, 1.06) | 1.03 (1.00, 1.07) | 1.10 (1.02, 1.20) * | 1.09 (1.01, 1.17) * |
| Secondary (grade 6-10) | 1.03 (1.00, 1.06) | 1.04 (1.01, 1.08) * | 1.27 (1.18, 1.37) *** | 1.28 (1.18, 1.38) *** |
| Higher secondary & above | 1.07 (1.03, 1.11) *** | 1.11 (1.06, 1.15) *** | 1.57 (1.43, 1.72) *** | 1.41 (1.28, 1.55) *** |
| **Occupation** |  | | | |
| Not working/homemaker | Ref | Ref | Ref | Ref |
| Working | 1.00 (0.96, 1.05) | N/A | 1.25 (1.16, 1.35) *** | 1.00 (0.93, 1.08) |
| **Wealth quintile** |  | | | |
| Poorest | Ref | Ref | Ref | Ref |
| Poorer | 1.02 (0.98, 1.07) | 1.04 (1.00, 1.08) | 1.03 (0.91, 1.15) | 1.00 (0.91, 1.11) |
| Middle | 1.00 (0.96, 1.04) | 1.01 (0.97, 1.05) | 1.23 (1.11, 1.38) *** | 1.12 (1.02, 1.23) * |
| Richer | 1.04 (1.00, 1.08) * | 1.02 (0.98, 1.06) | 1.63 (1.48, 1.79) *** | 1.17 (1.07, 1.29) ** |
| Richest | 1.09 (1.05, 1.13) *** | 1.04 (1.00, 1.09) * | 1.96 (1.79, 2.15) *** | 1.18 (1.07, 1.30) ** |
| **Physical activity** |  | | | |
| >=150 Minutes/week | Ref | Ref | Ref | Ref |
| <150 Minutes/week | 1.05(1.02, 1.07) ** | 1.02 (0.99, 1.06) | 1.40 (1.31, 1.49) *** | 0.99 (0.9, 1.09) |
| **Fruits and vegetables intake** |  | | | |
| >= 5 servings/day | Ref | Ref | Ref | Ref |
| <5 servings/day | 1.01 (0.98, 1.04) | N/A | 1.34 (1.20, 1.49) *** | 1.11 (1.00, 1.23) * |
| **Sedentary time** |  | | | |
| <= 7 hours | Ref |  | Ref |  |
| >7hours | 1.05 (1.03, 1.08) *** | 1.01 (0.99, 1.04) | 1.26 (1.19, 1.34) *** | 1.09 (1.02, 1.17) * |
| **Duration of watching TV** |  | | | |
| <=4 hours | Ref |  | Ref |  |
| >4hours | 1.05 (1.01, 1.10) ** | 0.99 (0.94, 1.04) | 1.49 (1.39, 1.61) *** | 1.00 (0.88, 1.13) |
| **Current smoker** |  | | | |
| No | Ref |  | Ref |  |
| Yes | 1.16 (1.13, 1.18) *** | 1.16 (1.13, 1.18) *** | 1.09 (0.82, 1.45) | N/A |
| **Body mass Index (BMI)** |  | | | |
| Underweight | Ref |  | Ref |  |
| Normal | 1 (0.97, 1.04) | N/A | 1.07 (0.95, 1.21) | 1.05 (0.94, 1.16) |
| Overweight and/or obese | 1.02 (0.99, 1.06) |  | 1.38 (1.23, 1.55) *** | 1.15 (1.04, 1.27) ** |
| **Hypertension** |  | | | |
| Non-hypertensive | Ref |  | Ref |  |
| Hypertensive | 0.98 (0.95, 1.01) | N/A | 1.15 (1.08, 1.22) *** | 1.06 (1, 1.13) * |
| **Self -reported heart disease** |  | | | |
| No | Ref |  | Ref |  |
| Yes | 0.99 (0.94, 1.04) | N/A | 0.99 (0.89, 1.09) | N/A |
| **Self- reported asthma** |  | | | |
| No | Ref |  | Ref |  |
| Yes | 1.00 (0.95, 1.06) | N/A | 1.12 (1.01, 1.25) * | 1.14 (1.04, 1.26) ** |
| **Self- reported diabetes** |  | | | |
| No | Ref |  | Ref |  |
| Yes | 0.83 (0.75, 0.91) *** | 0.80 (0.73, 0.88) *** | 1.07 (0.96, 1.20) | N/A |

*p<0.05; **p<0.01; ***p<0.001

^a^Hindu, Christian, Buddhist

^b^Never married, separated, divorced, widowed
