# Supplementary material for: Unhealthy food consumption among 20–59 years old adults in Bangladesh: Findings from a nationally representative cross-sectional survey
Source: PLoS One. 2025 Dec 2;20(12):e0336984. doi: 10.1371/journal.pone.0336984 (PMC12671833; doi:10.1371/journal.pone.0336984)
Supplement: S8 Table — (DOCX) [file pone.0336984.s008.docx]

S8 Table. Crude prevalence ratios (CPR) and adjusted prevalence ratios (APR) of the factors of SFS consumption among men and women (Bonferroni corrected)

| **Variables** | **Men** | | | | | | | | **Women** | | | | | | | |
| --- | --- | --- | --- | --- | --- | --- | --- | --- | --- | --- | --- | --- | --- | --- | --- | --- |
|  | **CPR** | **SE** | **P value** | **95% CI** | **APR** | **SE** | **P value** | **95% CI** | **CPR** | **SE** | **P value** | **95% CI** | **APR** | **SE** | **P value** | **95% CI** |
| **Age in years** |  |  |  |  |  |  |  |  |  |  |  |  |  |  |  |  |
| 20-29 | 1.53 | 0.06 | <0.001 | 1.41, 1.67 | 1.39 | 0.06 | <0.001 | 1.27, 1.53 | 1.74 | 0.14 | <0.001 | 1.47, 2.05 | 1.36 | 0.12 | 0.001 | 1.13, 1.63 |
| 30-39 | 1.33 | 0.06 | <0.001 | 1.22, 1.45 | 1.23 | 0.05 | <0.001 | 1.12, 1.34 | 1.52 | 0.12 | <0.001 | 1.28, 1.80 | 1.25 | 0.1 | 0.019 | 1.05, 1.49 |
| 40-49 | 1.19 | 0.05 | <0.001 | 1.09, 1.31 | 1.15 | 0.05 | <0.001 | 1.05, 1.26 | 1.26 | 0.11 | 0.025 | 1.04, 1.51 | 1.15 | 0.1 | 0.281 | 0.96, 1.39 |
| 50-59 | Ref |  |  |  |  |  |  |  | Ref |  |  |  |  |  |  |  |
| **Division** |  |  |  |  |  |  |  |  |  |  |  |  |  |  |  |  |
| Dhaka | 0.78 | 0.04 | <0.001 | 0.69, 0.88 | 0.77 | 0.04 | <0.001 | 0.68,0.87 | 0.87 | 0.06 | 0.352 | 0.73, 1.04 | 0.81 | 0.06 | 0.039 | 0.67, 0.98 |
| Chittagong | 1.14 | 0.04 | <0.001 | 1.05, 1.24 | 1.12 | 0.04 | <0.001 | 1.03,1.22 | 1.20 | 0.07 | 0.022 | 1.03, 1.40 | 1.16 | 0.07 | 0.143 | 0.99, 1.35 |
| Rajshahi | 0.86 | 0.04 | 0.003 | 0.78, 0.95 | 0.88 | 0.04 | 0.020 | 0.79,0.98 | 0.82 | 0.06 | 0.055 | 0.68, 0.98 | 0.85 | 0.06 | 0.186 | 0.71, 1.02 |
| Khulna | Ref |  |  |  |  |  |  |  | Ref |  |  |  |  |  |  |  |
| Barisal | 0.98 | 0.04 | 1.000 | 0.88, 1.09 | 0.98 | 0.04 | 1.000 | 0.88,1.09 | 1.26 | 0.08 | 0.005 | 1.07, 1.48 | 1.27 | 0.09 | 0.005 | 1.07, 1.52 |
| Sylhet | 0.86 | 0.04 | 0.020 | 0.76, 0.97 | 0.88 | 0.04 | 0.051 | 0.77,0.99 | 0.78 | 0.07 | 0.032 | 0.63, 0.97 | 0.82 | 0.07 | 0.205 | 0.66, 1.02 |
| Rangpur | 0.83 | 0.04 | <0.001 | 0.75, 0.92 | 0.86 | 0.04 | 0.004 | 0.77,0.96 | 0.60 | 0.05 | <0.001 | 0.49, 0.74 | 0.63 | 0.05 | <0.001 | 0.51, 0.78 |
| Mymensingh | 0.69 | 0.03 | <0.001 | 0.61, 0.78 | 0.68 | 0.03 | <0.001 | 0.6,0.77 | 0.56 | 0.05 | <0.001 | 0.46, 0.70 | 0.57 | 0.05 | <0.001 | 0.46, 0.71 |
| **Area** |  |  |  |  |  |  |  |  |  |  |  |  |  |  |  |  |
| Urban | 1.13 | 0.03 | <0.001 | 1.07, 1.20 | 1.11 | 0.04 | 0.012 | 1.03,1.19 | 1.43 | 0.06 | <0.001 | 1.31, 1.56 | 1.19 | 0.07 | 0.009 | 1.06, 1.35 |
| Rural | Ref |  |  |  |  |  |  |  | Ref |  |  |  |  |  |  |  |
| Slum | 1.13 | 0.04 | <0.001 | 1.06, 1.21 | 1.12 | 0.04 | 0.002 | 1.05,1.2 | 1.25 | 0.07 | <0.001 | 1.12, 1.40 | 1.18 | 0.07 | 0.010 | 1.05, 1.33 |
| **Religion** |  |  |  |  |  |  |  |  |  |  |  |  |  |  |  |  |
| Islam | Ref |  |  |  |  |  |  |  | Ref |  |  |  |  |  |  |  |
| Others^a^ | 1.02 | 0.03 | 0.636 | 0.95, 1.08 | N/A |  |  |  | 1.03 | 0.06 | 0.587 | 0.92, 1.15 | N/A |  |  |  |
| **Marital status** |  |  |  |  |  |  |  |  |  |  |  |  |  |  |  |  |
| Currently married | Ref |  |  |  |  |  |  |  | Ref |  |  |  |  |  |  |  |
| Others^b^ | 1.22 | 0.03 | <0.001 | 1.16, 1.29 | 1.03 | 0.04 | 0.392 | 0.96, 1.10 | 0.84 | 0.06 | 0.023 | 0.72, 0.98 | 0.88 | 0.07 | 0.096 | 0.76, 1.02 |
| **Education** |  |  |  |  |  |  |  |  |  |  |  |  |  |  |  |  |
| No formal education | Ref |  |  |  |  |  |  |  | Ref |  |  |  |  |  |  |  |
| Primary | 1.26 | 0.04 | <0.001 | 1.17, 1.35 | 1.14 | 0.04 | 0.002 | 1.06, 1.23 | 1.36 | 0.08 | <0.001 | 1.2,1.54 | 1.2 | 0.07 | 0.008 | 1.06, 1.36 |
| Secondary | 1.35 | 0.05 | <0.001 | 1.26, 1.45 | 1.22 | 0.04 | 0.002 | 1.13, 1.31 | 1.67 | 0.09 | <0.001 | 1.49,1.87 | 1.38 | 0.08 | <0.001 | 1.22, 1.57 |
| Higher secondary & above | 1.38 | 0.05 | <0.001 | 1.28, 1.49 | 1.28 | 0.06 | 0.002 | 1.16, 1.40 | 2.04 | 0.13 | <0.001 | 1.77,2.34 | 1.63 | 0.12 | <0.001 | 1.39, 1.92 |
| **Occupation** |  |  |  |  |  |  |  |  |  |  |  |  |  |  |  |  |
| Not working | Ref |  |  |  |  |  |  |  | Ref |  |  |  |  |  |  |  |
| Working | 0.9 | 0.04 | 0.006 | 0.83, 0.97 | 1.07 | 0.05 | 0.166 | 0.97, 1.17 | 1.13 | 0.07 | 0.038 | 1.01, 1.27 | 1.12 | 0.07 | 0.055 | 1.00, 1.26 |
| **Wealth quintile** |  |  |  |  |  |  |  |  |  |  |  |  |  |  |  |  |
| Lowest | Ref |  |  |  |  |  |  |  | Ref |  |  |  |  |  |  |  |
| Second | 1.11 | 0.04 | 0.026 | 1.02, 1.22 | 1.12 | 0.04 | 0.013 | 1.03, 1.22 | 1.16 | 0.08 | 0.135 | 0.99, 1.35 | 1.15 | 0.08 | 0.139 | 0.99, 1.34 |
| Middle | 1.14 | 0.04 | 0.002 | 1.05, 1.25 | 1.11 | 0.04 | 0.015 | 1.02, 1.21 | 1.22 | 0.08 | 0.017 | 1.04, 1.42 | 1.15 | 0.08 | 0.156 | 0.99, 1.35 |
| Fourth | 1.14 | 0.04 | 0.004 | 1.04, 1.24 | 1.08 | 0.04 | 0.154 | 0.99, 1.18 | 1.38 | 0.09 | <0.001 | 1.19, 1.59 | 1.19 | 0.08 | 0.041 | 1.02, 1.38 |
| Highest | 1.21 | 0.05 | <0.001 | 1.11, 1.32 | 1.10 | 0.04 | 0.063 | 1.01, 1.20 | 1.69 | 0.1 | <0.001 | 1.47, 1.94 | 1.33 | 0.09 | <0.001 | 1.14, 1.57 |
| **IPA** |  |  |  |  |  |  |  |  |  |  |  |  |  |  |  |  |
| >=150 Minutes/week | Ref |  |  |  |  |  |  |  | Ref |  |  |  |  |  |  |  |
| <150 Minutes/week | 1.06 | 0.03 | 0.022 | 1.01, 1.12 | 0.96 | 0.03 | 0.174 | 0.90, 1.02 | 1.37 | 0.07 | <0.001 | 1.25, 1.51 | 1.11 | 0.07 | 0.117 | 0.97, 1.26 |
| **Fruits and vegetables intake** |  |  |  |  |  |  |  |  |  |  |  |  |  |  |  |  |
| >= 5 servings/day | Ref |  |  |  |  |  |  |  | Ref |  |  |  |  |  |  |  |
| <5 servings/day | 0.96 | 0.03 | 0.111 | 0.91, 1.01 | 0.94 | 0.03 | 0.049 | 0.88, 1.00 | 0.89 | 0.05 | 0.038 | 0.79, 0.99 | 0.81 | 0.05 | <0.001 | 0.72, 0.91 |
| **Sedentary time** |  |  |  |  |  |  |  |  |  |  |  |  |  |  |  |  |
| <= 7 hours | Ref |  |  |  |  |  |  |  | Ref |  |  |  |  |  |  |  |
| >7hours | 1.08 | 0.03 | 0.002 | 1.03, 1.14 | 1.01 | 0.03 | 0.615 | 0.96, 1.08 | 1.17 | 0.05 | <0.001 | 1.07, 1.28 | 1.00 | 0.05 | 0.975 | 0.90, 1.11 |
| **Duration of watching TV** |  |  |  |  |  |  |  |  |  |  |  |  |  |  |  |  |
| <=4 hours | Ref |  |  |  |  |  |  |  | Ref |  |  |  |  |  |  |  |
| >4hours | 1.16 | 0.04 | <0.001 | 1.08, 1.25 | 0.93 | 0.05 | 0.164 | 0.83, 1.03 | 1.32 | 0.08 | <0.001 | 1.17, 1.48 | 0.85 | 0.08 | 0.09 | 0.71, 1.03 |
| **Current smoker** |  |  |  |  |  |  |  |  |  |  |  |  |  |  |  |  |
| No | Ref |  |  |  |  |  |  |  | Ref |  |  |  |  |  |  |  |
| Yes | 1.04 | 0.02 | 0.068 | 1.00, 1.09 | 1.10 | 0.03 | <0.001 | 1.05, 1.15 | 0.80 | 0.2 | 0.364 | 0.49, 1.30 | N/A |  |  |  |
| **Body Mass Index (BMI)** |  |  |  |  |  |  |  |  |  |  |  |  |  |  |  |  |
| Underweight | Ref |  |  |  |  |  |  |  | Ref |  |  |  |  |  |  |  |
| Normal | 1.02 | 0.04 | 1.000 | 0.96, 1.10 | 1.02 | 0.03 | 1.199 | 0.95, 1.09 | 1.07 | 0.08 | 0.784 | 0.07, 0.08 | 0.95 | 0.07 | 1.000 | 0.82, 1.10 |
| Overweight/Obese | 1.09 | 0.04 | 0.030 | 1.02, 1.17 | 1.06 | 0.04 | 0.274 | 0.98, 1.14 | 1.24 | 0.09 | 0.007 | 0.21, 0.07 | 0.98 | 0.07 | 1.000 | 0.85, 1.13 |
| **Self -reported HTN** |  |  |  |  |  |  |  |  |  |  |  |  |  |  |  |  |
| Non-hypertensive | Ref |  |  |  |  |  |  |  | Ref |  |  |  |  |  |  |  |
| Hypertensive | 0.93 | 0.03 | 0.013 | 0.87, 0.98 | 0.97 | 0.03 | 0.275 | 0.91, 1.03 | 0.98 | 0.05 | 0.099 | 0.89, 1.07 | 1.03 | 0.05 | 0.516 | 0.94, 1.13 |
| **Self -reported heart disease** |  |  |  |  |  |  |  |  |  |  |  |  |  |  |  |  |
| No | Ref |  |  |  |  |  |  |  | Ref |  |  |  |  |  |  |  |
| Yes | 0.86 | 0.05 | 0.005 | 0.77, 0.96 | 0.95 | 0.05 | 0.369 | 0.86, 1.06 | 0.96 | 0.07 | 0.539 | 0.83, 1.10 | N/A |  |  |  |
| **Self- reported asthma** |  |  |  |  |  |  |  |  |  |  |  |  |  |  |  |  |
| No | Ref |  |  |  |  |  |  |  | Ref |  |  |  |  |  |  |  |
| Yes | 0.92 | 0.05 | 0.163 | 0.83, 1.03 | 0.99 | 0.05 | 0.888 | 0.89, 1.10 | 1.07 | 0.08 | 0.359 | 0.92, 1.24 | N/A |  |  |  |
| **Self- reported diabetes** |  |  |  |  |  |  |  |  |  |  |  |  |  |  |  |  |
| No | Ref |  |  |  |  |  |  |  | Ref |  |  |  |  |  |  |  |
| Yes | 0.85 | 0.06 | 0.022 | 0.74, 0.98 | 0.92 | 0.07 | 0.224 | 0.80, 1.06 | 0.93 | 0.08 | 0.373 | 0.78, 1.10 | N/A |  |  |  |

^a^Hindu, Christian, Buddhist together

^b^Never married, separated, divorced, widowed
