# Supplementary material for: Unhealthy food consumption among 20–59 years old adults in Bangladesh: Findings from a nationally representative cross-sectional survey
Source: PLoS One. 2025 Dec 2;20(12):e0336984. doi: 10.1371/journal.pone.0336984 (PMC12671833; doi:10.1371/journal.pone.0336984)
Supplement: S9 Table — (DOCX) [file pone.0336984.s009.docx]

S9 Table. Crude prevalence ratios (CPR) and adjusted prevalence ratios (APR) of the factors of SS consumption among men and women (Bonferroni corrected)

| **Variables** | **Men** | | | | | | | | **Women** | | | | | | | |
| --- | --- | --- | --- | --- | --- | --- | --- | --- | --- | --- | --- | --- | --- | --- | --- | --- |
|  | **CPR** | **SE** | **P value** | **95% CI** | **APR** | **SE** | **95% CI** | **P value** | **CPR** | **SE** | **95% CI** | **P value** | **APR** | **SE** | **P value** | **95% CI** |
| **Age in years** |  |  |  |  |  |  |  |  |  |  |  |  |  |  |  |  |
| 20-29 | 1.14 | 0.03 | <0.001 | 1.08, 1.20 | 1.06 | 0.03 | 1.00, 1.12 | 0.068 | 1.19 | 0.05 | 1.10, 1.29 | 0.000 | 1.03 | 0.04 | 1.000 | 0.95, 1.13 |
| 30-39 | 1.07 | 0.03 | 0.023 | 1.01, 1.12 | 1.03 | 0.02 | 0.98, 1.08 | 0.767 | 1.12 | 0.04 | 1.03, 1.21 | 0.013 | 1.00 | 0.04 | 1.000 | 0.92, 1.09 |
| 40-49 | 1.04 | 0.03 | 0.464 | 0.98, 1.10 | 1.02 | 0.03 | 0.97, 1.08 | 1.255 | 1.08 | 0.05 | 0.99, 1.18 | 0.224 | 1.01 | 0.04 | 1.000 | 0.93, 1.11 |
| 50-59 | Ref |  |  |  |  |  |  |  | Ref |  |  |  |  |  |  |  |
| **Division** |  |  |  |  |  |  |  |  |  |  |  |  |  |  |  |  |
| Dhaka | 0.91 | 0.03 | 0.005 | 0.85, 0.97 | 0.89 | 0.03 | 0.83, 0.96 | <0.001 | 0.89 | 0.03 | 0.81, 0.98 | 0.022 | 0.87 | 0.03 | 0.003 | 0.79, 0.96 |
| Chittagong | 1.00 | 0.02 | 1.000 | 0.94, 1.05 | 0.98 | 0.02 | 0.92, 1.04 | 1.000 | 1.06 | 0.03 | 0.98, 1.15 | 0.517 | 1.02 | 0.03 | 1.000 | 0.95, 1.11 |
| Rajshahi | 0.77 | 0.02 | <0.001 | 0.71, 0.84 | 0.76 | 0.02 | 0.70, 0.82 | <0.001 | 0.76 | 0.03 | 0.68, 0.84 | 0.000 | 0.76 | 0.03 | <0.001 | 0.69, 0.85 |
| Khulna | Ref |  |  |  |  |  |  |  | Ref |  |  |  |  |  |  |  |
| Barisal | 1.02 | 0.02 | 1.000 | 0.96, 1.08 | 0.98 | 0.02 | 0.92, 1.04 | 1.000 | 0.99 | 0.04 | 0.90, 1.09 | 1.000 | 1.00 | 0.04 | 1.000 | 0.91, 1.09 |
| Sylhet | 1.05 | 0.02 | 0.455 | 0.99, 1.11 | 1.01 | 0.03 | 0.95, 1.07 | 1.000 | 1.13 | 0.04 | 1.05, 1.23 | 0.001 | 1.18 | 0.04 | <0.001 | 1.08, 1.29 |
| Rangpur | 0.82 | 0.02 | <0.001 | 0.76, 0.88 | 0.79 | 0.02 | 0.73, 0.85 | <0.001 | 0.68 | 0.03 | 0.60, 0.76 | 0.000 | 0.67 | 0.03 | <0.001 | 0.60, 0.75 |
| Mymensingh | 0.88 | 0.02 | <0.001 | 0.83, 0.95 | 0.86 | 0.02 | 0.80, 0.92 | <0.001 | 0.74 | 0.03 | 0.66, 0.82 | 0.000 | 0.76 | 0.03 | <0.001 | 0.68, 0.84 |
| **Area** |  |  |  |  |  |  |  |  |  |  |  |  |  |  |  |  |
| Urban | 1.00 | 0.02 | 1.000 | 0.96, 1.04 | 0.99 | 0.02 | 0.95, 1.04 | 1.000 | 1.19 | 0.03 | 1.14, 1.25 | 0.000 | 1.02 | 0.03 | 0.809 | 0.97, 1.09 |
| Rural | Ref |  |  |  |  |  |  |  | Ref |  |  |  |  |  |  |  |
| Slum | 1.05 | 0.02 | 0.079 | 1.00, 1.09 | 1.04 | 0.02 | 0.99, 1.09 | 0.167 | 1.06 | 0.03 | 0.99, 1.13 | 0.157 | 1.00 | 0.03 | 1.000 | 0.94, 1.07 |
| **Religion** |  |  |  |  |  |  |  |  |  |  |  |  |  |  |  |  |
| Islam | Ref |  |  |  |  |  |  |  | Ref |  |  |  |  |  |  |  |
| Others^a^ | 1.06 | 0.02 | 0.004 | 1.02, 1.10 | 0.99 | 0.02 | 0.95, 1.03 | 0.597 | 1.15 | 0.03 | 1.09, 1.21 | <0.001 | 1.03 | 0.03 | 0.287 | 0.98, 1.09 |
| **Marital status** |  |  |  |  |  |  |  |  |  |  |  |  |  |  |  |  |
| Currently married | Ref |  |  |  |  |  |  |  | Ref |  |  |  |  |  |  |  |
| Others^b^ | 1.10 | 0.02 | <0.001 | 1.06, 1.14 | 1.02 | 0.02 | 0.98, 1.07 | 0.299 | 0.97 | 0.04 | 0.90, 1.05 | 0.487 | N/A |  |  |  |
| **Education** |  |  |  |  |  |  |  |  |  |  |  |  |  |  |  |  |
| No formal education | Ref |  |  |  |  |  |  |  | Ref |  |  |  |  |  |  |  |
| Primary | 1.09 | 0.02 | <0.001 | 1.05, 1.15 | 1.07 | 0.02 | 1.02, 1.12 | 0.008 | 1.11 | 0.03 | 1.04, 1.19 | 0.002 | 1.07 | 0.03 | 0.075 | 1.00, 1.15 |
| Secondary | 1.12 | 0.02 | <0.001 | 1.07, 1.18 | 1.10 | 0.02 | 1.05, 1.15 | <0.001 | 1.27 | 0.04 | 1.20, 1.35 | <0.001 | 1.2 | 0.04 | <0.001 | 1.13, 1.29 |
| Higher secondary & above | 1.18 | 0.03 | <0.001 | 1.13, 1.24 | 1.18 | 0.03 | 1.12, 1.25 | <0.001 | 1.46 | 0.05 | 1.37, 1.57 | <0.001 | 1.39 | 0.05 | <0.001 | 1.28, 1.51 |
| **Occupation** |  |  |  |  |  |  |  |  |  |  |  |  |  |  |  |  |
| Not working | Ref |  |  |  |  |  |  |  | Ref |  |  |  |  |  |  |  |
| Working | 0.97 | 0.03 | 0.334 | 0.92, 1.03 | N/A |  |  |  | 1.04 | 0.03 | 0.97, 1.10 | 0.283 | N/A |  |  |  |
| **Wealth quintile** |  |  |  |  |  |  |  |  |  |  |  |  |  |  |  |  |
| Lowest | Ref |  |  |  |  |  |  |  | Ref |  |  |  |  |  |  |  |
| Second | 1.03 | 0.03 | 1.000 | 0.97, 1.08 | 1.02 | 0.02 | 0.97, 1.08 | 1.000 | 1.09 | 0.04 | 1.00, 1.18 | 0.131 | 1.06 | 0.04 | 0.360 | 0.98, 1.16 |
| Middle | 1.02 | 0.03 | 1.000 | 0.97, 1.08 | 1.01 | 0.02 | 0.96, 1.07 | 1.000 | 1.18 | 0.04 | 1.08, 1.28 | <0.001 | 1.12 | 0.04 | 0.008 | 1.03, 1.22 |
| Fourth | 1.04 | 0.03 | 0.426 | 0.98, 1.10 | 1.05 | 0.03 | 0.99, 1.11 | 0.195 | 1.26 | 0.04 | 1.16, 1.36 | <0.001 | 1.17 | 0.04 | <0.001 | 1.08, 1.27 |
| Highest | 1.09 | 0.03 | 0.001 | 1.03, 1.15 | 1.08 | 0.03 | 1.02, 1.14 | 0.006 | 1.36 | 0.05 | 1.26, 1.47 | <0.001 | 1.2 | 0.05 | <0.001 | 1.10, 1.30 |
| **IPA** |  |  |  |  |  |  |  |  |  |  |  |  |  |  |  |  |
| >=150 Minutes/week | Ref |  |  |  |  |  |  |  | Ref |  |  |  |  |  |  |  |
| <150 Minutes/week | 0.92 | 0.02 | <0.001 | 0.89, 0.96 | 0.92 | 0.02 | 0.89, 0.96 | <0.001 | 1.03 | 0.03 | 0.97, 1.09 | 0.333 | N/A |  |  |  |
| **Fruits and vegetables intake** |  |  |  |  |  |  |  |  |  |  |  |  |  |  |  |  |
| >= 5 servings/day | Ref |  |  |  |  |  |  |  | Ref |  |  |  |  |  |  |  |
| <5 servings/day | 1.05 | 0.02 | 0.016 | 1.01, 1.09 | 1.04 | 0.02 | 0.99, 1.08 | 0.094 | 0.99 | 0.03 | 0.93, 1.05 | 0.693 | N/A |  |  |  |
| **Sedentary time** |  |  |  |  |  |  |  |  |  |  |  |  |  |  |  |  |
| <= 7 hours | Ref |  |  |  |  |  |  |  | Ref |  |  |  |  |  |  |  |
| >7hours | 0.98 | 0.02 | 0.377 | 0.95, 1.02 | N/A |  |  |  | 1.05 | 0.03 | 1.00, 1.10 | 0.049 | 0.98 | 0.03 | 0.538 | 0.94, 1.04 |
| **Duration of watching TV** |  |  |  |  |  |  |  |  |  |  |  |  |  |  |  |  |
| <=4 hours | Ref |  |  |  |  |  |  |  | Ref |  |  |  |  |  |  |  |
| >4hours | 0.91 | 0.03 | 0.005 | 0.85, 0.97 | 0.92 | 0.04 | 0.84, 1.00 | 0.041 | 0.99 | 0.04 | 0.91, 1.07 | 0.735 | N/A |  |  |  |
| **Current smoker** |  |  |  |  |  |  |  |  |  |  |  |  |  |  |  |  |
| No | Ref |  |  |  |  |  |  |  | Ref |  |  |  |  |  |  |  |
| Yes | 1.03 | 0.02 | 0.101 | 1.00, 1.06 | 1.05 | 0.02 | 1.02, 1.08 | 0.001 | 0.92 | 0.92 | 0.72, 1.18 | 0.534 | N/A |  |  |  |
| **Body Mass Index (BMI)** |  |  |  |  |  |  |  |  |  |  |  |  |  |  |  |  |
| Underweight | Ref |  |  |  |  |  |  |  | Ref |  |  |  |  |  |  |  |
| Normal | 1.00 | 0.02 | 1.000 | 0.95,1.04 | N/A |  |  |  | 1.03 | 0.04 | 0.95, 1.11 | 1.000 | 1.01 | 0.04 | 1.000 | 0.93, 1.10 |
| Overweight/Obese | 1.01 | 0.02 | 1.000 | 0.96,1.05 |  |  |  |  | 1.16 | 0.05 | 1.07, 1.25 | <0.001 | 1.08 | 0.04 | 0.095 | 1.00, 1.17 |
| **Self -reported HTN** |  |  |  |  |  |  |  |  |  |  |  |  |  |  |  |  |
| Non-hypertensive | Ref |  |  |  |  |  |  |  | Ref |  |  |  |  |  |  |  |
| Hypertensive | 1.01 | 0.02 | 0.598 | 0.97, 1.05 | N/A |  |  |  | 1.05 | 0.03 | 1.00, 1.10 | 0.03 | 1.05 | 0.03 | 0.072 | 1.00, 1.10 |
| **Self -reported heart disease** |  |  |  |  |  |  |  |  |  |  |  |  |  |  |  |  |
| No | Ref |  |  |  |  |  |  |  | Ref |  |  |  |  |  |  |  |
| Yes | 0.99 | 0.03 | 0.699 | 0.93, 1.05 | N/A |  |  |  | 0.99 | 0.04 | 0.92, 1.07 | 0.809 | N/A |  |  |  |
| **Self- reported asthma** |  |  |  |  |  |  |  |  |  |  |  |  |  |  |  |  |
| No | Ref |  |  |  |  |  |  |  | Ref |  |  |  |  |  |  |  |
| Yes | 0.99 | 0.03 | 0.782 | 0.93, 1.06 | N/A |  |  |  | 0.98 | 0.04 | 0.90, 1.07 | 0.671 | N/A |  |  |  |
| **Self- reported diabetes** |  |  |  |  |  |  |  |  |  |  |  |  |  |  |  |  |
| No | Ref |  |  |  |  |  |  |  | Ref |  |  |  |  |  |  |  |
| Yes | 0.89 | 0.04 | 0.018 | 0.81, 0.98 | 0.91 | 0.04 | 0.83, 1.00 | 0.061 | 1.02 | 0.04 | 0.94, 1.11 | 0.667 | N/A |  |  |  |

^a^Hindu, Christian, Buddhist together

^b^Never married, separated, divorced, widowed
