# Supplementary material for: Unhealthy food consumption among 20–59 years old adults in Bangladesh: Findings from a nationally representative cross-sectional survey
Source: PLoS One. 2025 Dec 2;20(12):e0336984. doi: 10.1371/journal.pone.0336984 (PMC12671833; doi:10.1371/journal.pone.0336984)
Supplement: S10 Table — (DOCX) [file pone.0336984.s010.docx]

S10 Table. Crude prevalence ratios (CPR) and adjusted prevalence ratios (APR) of the factors of SSBs consumption among men and women (Bonferroni corrected)

| **Variables** | **Men** | | | | | | | | **Women** | | | | | | | |
| --- | --- | --- | --- | --- | --- | --- | --- | --- | --- | --- | --- | --- | --- | --- | --- | --- |
|  | **CPR** | **SE** | **p value** | **95% CI** | **APR** | **SE** | **P value** | **95% CI** | **CPR** | **SE** | **P value** | **95% CI** | **APR** | **SE** | **P value** | **95% CI** |
| **Age in years** |  |  |  |  |  |  |  |  |  |  |  |  |  |  |  |  |
| 20-29 | 1.01 | 0.02 | 1.000 | 0.97, 1.06 | 0.97 | 0.02 | 0.466 | 0.93, 1.01 | 1.15 | 0.06 | 0.026 | 1.03, 1.29 | 0.92 | 0.05 | 0.414 | 0.82, 1.04 |
| 30-39 | 1.04 | 0.02 | 0.076 | 1.00, 1.09 | 1.01 | 0.02 | 1.000 | 0.97, 1.05 | 1.18 | 0.06 | 0.005 | 1.05, 1.32 | 1 | 0.05 | 1.000 | 0.90, 1.12 |
| 40-49 | 1.05 | 0.02 | 0.045 | 1.01, 1.09 | 1.03 | 0.02 | 0.507 | 0.99, 1.07 | 1.15 | 0.06 | 0.040 | 1.02, 1.30 | 1.06 | 0.05 | 0.667 | 0.95, 1.19 |
| 50-59 | Ref |  |  |  |  |  |  |  | Ref |  |  |  |  |  |  |  |
| **Division** |  |  |  |  |  |  |  |  |  |  |  |  |  |  |  |  |
| Dhaka | 1.08 | 0.03 | 0.080 | 1.00, 1.16 | 1.06 | 0.03 | 0.272 | 0.99, 1.15 | 2.3 | 0.19 | <0.001 | 1.87, 2.82 | 1.97 | 0.15 | <0.001 | 1.63, 2.38 |
| Chittagong | 1.22 | 0.03 | <0.001 | 1.15, 1.29 | 1.21 | 0.03 | <0.001 | 1.14, 1.28 | 3.78 | 0.29 | <0.001 | 3.14, 4.56 | 3.59 | 0.25 | <0.001 | 3.03, 4.26 |
| Rajshahi | 0.98 | 0.03 | 1.000 | 0.9, 1.06 | 0.98 | 0.03 | 1.000 | 0.91, 1.06 | 1.56 | 0.14 | <0.001 | 1.25, 1.95 | 1.6 | 0.13 | <0.001 | 1.32, 1.96 |
| Khulna | Ref |  |  |  |  |  |  |  | Ref |  |  |  |  |  |  |  |
| Barisal | 1.22 | 0.03 | <0.001 | 1.15, 1.30 | 1.22 | 0.03 | <0.001 | 1.15, 1.30 | 2.73 | 0.23 | <0.001 | 2.23, 3.35 | 2.83 | 0.22 | <0.001 | 2.35, 3.42 |
| Sylhet | 1.23 | 0.03 | <0.001 | 1.15, 1.31 | 1.22 | 0.03 | <0.001 | 1.14, 1.30 | 4.12 | 0.31 | <0.001 | 3.42, 4.96 | 4.5 | 0.32 | <0.001 | 3.78, 5.35 |
| Rangpur | 1.14 | 0.03 | <0.001 | 1.06, 1.21 | 1.16 | 0.03 | <0.001 | 1.08, 1.24 | 1.80 | 0.16 | <0.001 | 1.45, 2.24 | 1.95 | 0.15 | <0.001 | 1.60, 2.37 |
| Mymensingh | 1.14 | 0.03 | <0.001 | 1.06, 1.22 | 1.13 | 0.03 | <0.001 | 1.06, 1.21 | 1.34 | 0.13 | 0.017 | 1.06, 1.69 | 1.4 | 0.12 | 0.001 | 1.13, 1.74 |
| **Area** |  |  |  |  |  |  |  |  |  |  |  |  |  |  |  |  |
| Urban | 1.14 | 0.01 | <0.001 | 1.11, 1.17 | 1.12 | 0.02 | <0.001 | 1.08, 1.16 | 2.04 | 0.06 | <0.001 | 1.92, 2.16 | 1.75 | 0.07 | <0.001 | 1.62, 1.89 |
| Rural | Ref |  |  |  |  |  |  |  | Ref |  |  |  |  |  |  |  |
| Slum | 1.1 | 0.02 | <0.001 | 1.07, 1.14 | 1.08 | 0.02 | <0.001 | 1.05, 1.12 | 1.92 | 0.07 | <0.001 | 1.80, 2.05 | 1.74 | 0.07 | <0.001 | 1.61, 1.88 |
| **Religion** |  |  |  |  |  |  |  |  |  |  |  |  |  |  |  |  |
| Islam | Ref |  |  |  |  |  |  |  | Ref |  |  |  |  |  |  |  |
| Others^a^ | 1.01 | 0.02 | 0.599 | 0.98, 1.04 | N/A |  |  |  | 1.22 | 0.05 | <0.001 | 1.14, 1.31 | 1.10 | 0.04 | 0.007 | 1.03, 1.17 |
| **Marital status** |  |  |  |  |  |  |  |  |  |  |  |  |  |  |  |  |
| Currently married | Ref |  |  |  |  |  |  |  | Ref |  |  |  |  |  |  |  |
| Others^b^ | 0.98 | 0.02 | 0.301 | 0.95, 1.02 | N/A |  |  |  | 1.17 | 0.05 | 0.001 | 1.07, 1.28 | 1.11 | 0.05 | 0.016 | 1.02, 1.21 |
| **Education** |  |  |  |  |  |  |  |  |  |  |  |  |  |  |  |  |
| No formal education | Ref |  |  |  |  |  |  |  | Ref |  |  |  |  |  |  |  |
| Primary | 1.02 | 0.02 | 0.454 | 0.99, 1.06 | 1.03 | 0.02 | 0.164 | 1.00, 1.07 | 1.1 | 0.05 | 0.059 | 1.01, 1.21 | 1.09 | 0.04 | 0.071 | 1.01, 1.18 |
| Secondary | 1.03 | 0.02 | 0.280 | 0.99, 1.07 | 1.04 | 0.02 | 0.059 | 1.00, 1.08 | 1.27 | 0.05 | <0.001 | 1.17, 1.38 | 1.28 | 0.05 | <0.001 | 1.18, 1.39 |
| Higher secondary & above | 1.07 | 0.02 | 0.001 | 1.03, 1.11 | 1.11 | 0.02 | <0.001 | 1.06, 1.16 | 1.57 | 0.07 | <0.001 | 1.42, 1.73 | 1.41 | 0.07 | <0.001 | 1.27, 1.56 |
| **Occupation** |  |  |  |  |  |  |  |  |  |  |  |  |  |  |  |  |
| Not working | Ref |  |  |  |  |  |  |  | Ref |  |  |  |  |  |  |  |
| Working | 1.00 | 0.02 | 0.902 | 0.96, 1.05 | N/A |  |  |  | 1.25 | 0.05 | <0.001 | 1.16, 1.35 | 1.00 | 0.04 | 0.972 | 0.93, 1.08 |
| **Wealth quintile** |  |  |  |  |  |  |  |  |  |  |  |  |  |  |  |  |
| Lowest | Ref |  |  |  |  |  |  |  | Ref |  |  |  |  |  |  |  |
| Second | 1.02 | 0.02 | 1.000 | 0.98, 1.07 | 1.04 | 0.02 | 0.207 | 0.99, 1.09 | 1.03 | 0.06 | 1.000 | 0.90, 1.17 | 1 | 0.05 | 1.000 | 0.90, 1.13 |
| Middle | 1 | 0.02 | 1.000 | 0.96, 1.05 | 1.01 | 0.02 | 1.000 | 0.96, 1.05 | 1.23 | 0.07 | 0.001 | 1.09, 1.40 | 1.12 | 0.06 | 0.081 | 1.00, 1.25 |
| Fourth | 1.04 | 0.02 | 0.176 | 1.10, 1.09 | 1.02 | 0.02 | 1.000 | 0.97, 1.06 | 1.63 | 0.08 | <0.001 | 1.46, 1.82 | 1.17 | 0.06 | 0.004 | 1.05, 1.30 |
| Highest | 1.09 | 0.02 | <0.001 | 1.04, 1.14 | 1.04 | 0.02 | 0.113 | 1.00, 1.09 | 1.96 | 0.09 | <0.001 | 1.77, 2.18 | 1.18 | 0.06 | 0.003 | 1.06, 1.32 |
| **IPA** |  |  |  |  |  |  |  |  |  |  |  |  |  |  |  |  |
| >=150 Minutes/week | Ref |  |  |  |  |  |  |  | Ref |  |  |  |  |  |  |  |
| <150 Minutes/week | 1.05 | 0.01 | 0.001 | 1.02, 1.07 | 1.02 | 0.02 | 0.126 | 0.99, 1.06 | 1.40 | 0.05 | <0.001 | 1.31, 1.49 | 0.99 | 0.05 | 0.889 | 0.90, 1.09 |
| **Fruits and vegetables intake** |  |  |  |  |  |  |  |  |  |  |  |  |  |  |  |  |
| >= 5 servings/day | Ref |  |  |  |  |  |  |  | Ref |  |  |  |  |  |  |  |
| <5 servings/day | 1.01 | 0.02 | 0.61 | 0.98, 1.04 | N/A |  |  |  | 1.34 | 0.08 | <0.001 | 1.20, 1.49 | 1.11 | 0.06 | 0.041 | 1.00, 1.23 |
| **Sedentary time** |  |  |  |  |  |  |  |  |  |  |  |  |  |  |  |  |
| <= 7 hours | Ref |  |  |  |  |  |  |  | Ref |  |  |  |  |  |  |  |
| >7hours | 1.05 | 0.01 | <0.001 | 1.03, 1.08 | 1.01 | 0.01 | 0.331 | 0.99, 1.04 | 1.26 | 0.04 | <0.001 | 1.19, 1.34 | 1.09 | 0.04 | 0.014 | 1.02, 1.17 |
| **Duration of watching TV** |  |  |  |  |  |  |  |  |  |  |  |  |  |  |  |  |
| <=4 hours | Ref |  |  |  |  |  |  |  | Ref |  |  |  |  |  |  |  |
| >4hours | 1.05 | 0.02 | 0.009 | 1.01, 1.10 | 0.99 | 0.03 | 0.729 | 0.94, 1.04 | 1.49 | 0.06 | <0.001 | 1.39, 1.61 | 1.00 | 0.06 | 0.977 | 0.88, 1.13 |
| **Current smoker** |  |  |  |  |  |  |  |  |  |  |  |  |  |  |  |  |
| No | Ref |  |  |  |  |  |  |  | Ref |  |  |  |  |  |  |  |
| Yes | 1.16 | 0.01 | <0.001 | 1.13, 1.18 | 1.16 | 0.01 | <0.001 | 1.13, 1.18 | 1.09 | 0.16 | 0.533 | 0.82, 1.45 | N/A |  |  |  |
| **Body Mass Index (BMI)** |  |  |  |  |  |  |  |  |  |  |  |  |  |  |  |  |
| Underweight | Ref |  |  |  |  |  |  |  | Ref |  |  |  |  |  |  |  |
| Normal | 1 | 0.02 | 1.000 | 0.97,1.04 | N/A |  |  |  | 1.07 | 0.07 | 0.543 | 0.95, 1.21 | 1.05 | 0.05 | 0.782 | 0.94, 1.16 |
| Overweight/Obese | 1.02 | 0.02 | 0.436 | 0.99,1.06 |  |  |  |  | 1.38 | 0.08 | <0.001 | 1.23, 1.55 | 1.15 | 0.06 | 0.010 | 1.04, 1.27 |
| **Self -reported HTN** |  |  |  |  |  |  |  |  |  |  |  |  |  |  |  |  |
| Non-hypertensive | Ref |  |  |  |  |  |  |  | Ref |  |  |  |  |  |  |  |
| Hypertensive | 0.98 | 0.02 | 0.242 | 0.95, 1.01 | N/A |  |  |  | 1.15 | 0.04 | <0.001 | 1.08, 1.22 | 1.06 | 0.03 | 0.044 | 1.00, 1.13 |
| **Self -reported heart disease** |  |  |  |  |  |  |  |  |  |  |  |  |  |  |  |  |
| No | Ref |  |  |  |  |  |  |  | Ref |  |  |  |  |  |  |  |
| Yes | 0.99 | 0.03 | 0.65 | 0.94, 1.04 | N/A |  |  |  | 0.99 | 0.05 | 0.795 | 0.89, 1.09 | N/A |  |  |  |
| **Self- reported asthma** |  |  |  |  |  |  |  |  |  |  |  |  |  |  |  |  |
| No | Ref |  |  |  |  |  |  |  | Ref |  |  |  |  |  |  |  |
| Yes | 1 | 0.03 | 0.982 | 0.95, 1.06 | N/A |  |  |  | 1.12 | 0.06 | 0.034 | 1.01, 1.25 | 1.14 | 0.05 | 0.005 | 1.04, 1.26 |
| **Self- reported diabetes** |  |  |  |  |  |  |  |  |  |  |  |  |  |  |  |  |
| No | Ref |  |  |  |  |  |  |  | Ref |  |  |  |  |  |  |  |
| Yes | 0.83 | 0.04 | <0.001 | 0.75, 0.91 | 0.80 | 0.04 | <0.001 | 0.73, 0.88 | 1.07 | 0.06 | 0.239 | 0.96, 1.20 | N/A |  |  |  |

^a^Hindu, Christian, Buddhist together

^b^Never married, separated, divorced, widowed
