# Supplementary material for: Unhealthy food consumption among 20–59 years old adults in Bangladesh: Findings from a nationally representative cross-sectional survey
Source: PLoS One. 2025 Dec 2;20(12):e0336984. doi: 10.1371/journal.pone.0336984 (PMC12671833; doi:10.1371/journal.pone.0336984)
Supplement: Attachment — Submitted filename: Response to Reviewers.docx [file pone.0336984.s015.docx]

**Manuscript ID: PONE-D-25-00015**

Manuscript title: Unhealthy food consumption among 20-59 years old adults in Bangladesh: findings from a nationally representative cross-sectional survey

**Additional Editor Comments**

1. Thank you for submitting your manuscript to PLOS ONE. We appreciate your contribution to addressing the pressing issue of unhealthy food consumption in Bangladesh using nationally representative data. After a careful evaluation by a peer reviewer and an editorial assessment, we have decided on your submission. Your manuscript addresses an important and timely topic in public health, offering valuable insights into dietary behaviors and their associated sociodemographic and clinical factors within the context of low- and middle-income countries. However, substantial revisions are required to enhance the scientific rigor, clarity, and interpretability of your study, and we are recommending a major revision at this stage.

**Response:** Thank you for your suggestion. We have revised the manuscript as per your recommendations. Please find the details below.

2. Several key concerns and required revisions need to be addressed. First, the binary classification of food consumption as weekly versus none lacks sensitivity in capturing habitual dietary behavior. We encourage you to justify this classification using supporting literature and consider including more granular frequency categories if available or acknowledge this limitation. Additionally, while your manuscript mentions model fitness, it is missing critical information. Please report variance inflation factors (VIFs) to assess multicollinearity and include goodness-of-fit statistics, such as the Pearson Chi-square or deviance, for your Poisson regression models.

**Response:** Thank you for your observation. We agree that categorizing food consumption simply as weekly versus none does not adequately reflect habitual dietary behavioral patterns. However, there are several papers that have used binary classification of food consumption for analysis (<https://pubmed.ncbi.nlm.nih.gov/35392287/>, <https://pubmed.ncbi.nlm.nih.gov/32029986/>, <https://pubmed.ncbi.nlm.nih.gov/12833112/>). Based on these studies, we developed this binary classification.

As per your suggestion, we have reported multicollinearity and goodness-of-fit statistics for all models considered for three food groups (savory and fried snacks, SFS; sweet snacks, SS; and sugar-sweetened beverages, SSBs), which can be found in supporting Tables S2, S3, and S4. Result section of the manuscript is also updated accordingly (page 13, lines 216-222). The text stated as follows:

***Multicollinearity and goodness-of-fit statistics:*** *Multicollinearity statistics for explanatory variables and goodness-of-fit statistics for SFS, SS, and SSBs, separately for adult men and women, are presented in Tables S2, S3, and S4, respectively. The mean VIF values are all less than 10 for all models considered, indicating no multicollinearity among the explanatory variables. Both deviance and Pearson Chi-square statistics showed large p values, indicating there is no lack of fit and suggesting that the Poisson regression models fit the data well.*

3. There is also the issue of multiple comparisons adjustment; given the number of predictors examined, failing to adjust for multiple testing increases the risk of Type I error. Consider applying a correction method like Bonferroni or FDR or to justify its omission explicitly. Moreover, the manuscript contains inconsistent terminology (e.g., "junk food" versus "unhealthy food") as well as various grammatical and structural issues. We strongly recommend professional English language editing to enhance readability and coherence.

**Response:** Thank you for your valuable comment. We used 18 predictors in the model and considered p-values <0.05 as statistically significant. Given this, there is less than a 5% chance that one predictor would be significant by chance. Moreover, for many of our factors, the p-value was <0.001. For this reason, we did not apply Bonferroni or FDR-based corrections.

In this manuscript, we used the term ‘unhealthy food’ to represent (savory and fried snacks, SFS; sweet snacks, SS; and sugar-sweetened beverages, SSBs). We used other terms, such as junk food and fast food, only in the introduction and discussion sections to introduce and explain the broader range of unhealthy foods mentioned by other authors and sources (<https://link.springer.com/article/10.1007/s10597-020-00672-x>, <https://pubmed.ncbi.nlm.nih.gov/38482184/>, <https://pubmed.ncbi.nlm.nih.gov/35816403/>). Regarding English editing we checked and edited the manuscript to correct grammatical errors.

4. The Discussion section could be further developed, as it restates results without adequate interpretation. We encourage you to situate your findings within established behavioral or theoretical frameworks, offer gender-sensitive interpretations, and provide clear policy implications and recommendations for targeted interventions. Additionally, the current limitations section is insufficient; you should discuss self-report bias and recall issues, as well as cross-sectional design limitations and the lack of data on portion size and caloric intake.

**Response:** We revised and enriched the whole discussion section as per your recommendation. We have added new text also (highlighted in yellow).

Thank you for your observation. We elaborated the section as you suggested in the strengths and limitations section. We added social desirability bias (please see line 428, page 30). We mentioned recall bias (please see line 430, page 30), the cross-sectional nature of the study (line 435, page 30), portion size (line 431, page 30), and the calorie intake issue (line 432, page 30).

5. Lastly, while your data availability statement claims full access, we encourage you to deposit the dataset in a public repository with a Digital Object Identifier (DOI) to promote transparency and reproducibility. The visual presentation of Tables 3 and 4 is also an area of concern; they are dense and would benefit from simplification. Consider adding figures such as bar charts or forest plots to enhance visual clarity.

**Response:** Thank you for your comment. Depositing the dataset in [Dryad](http://datadryad.org/) requires a data publishing fee, which we are unable to provide. However, we have shared the dataset as supporting information in both CSV and STATA formats.

As recommended, we have replaced Table 3 with a bar chart (now Fig 2) to present prevalence by gender and area of residence and moved the full table to the supplementary table (S1 Table). However, we would prefer to retain Table 4 in the main manuscript, as it presents both crude and adjusted prevalence ratios, which are central to our analysis and important for readers to interpret the key findings in context.

**Reviewer#1 comment**

1. Thank you for the opportunity to review this important and timely manuscript titled “Unhealthy food consumption among 20–59-year-old adults in Bangladesh: findings from a nationally representative cross-sectional survey.” The study addresses a critical public health issue using nationally representative data and provides helpful information regarding sociodemographic, behavioral, and clinical determinants of unhealthy food consumption. Major revisions are necessary to strengthen the scientific rigor, clarity, and overall contribution of this manuscript. Below are detailed comments organized according to the evaluation criteria:

**Response:** Thank you for your comment. We have revised the manuscript based on your suggestions. Please find the details below.

2. The simple yes-or-no classification of "weekly consumption" makes dietary behavior too basic and should either be explained better or changed to a more detailed measure that shows how often or how much is eaten.

**Response:** Thank you for your observation. We agree that categorizing food consumption simply as weekly versus none does not adequately reflect habitual dietary behavioral patterns. However, there are several papers that have used binary classification of food consumption for analysis (<https://pubmed.ncbi.nlm.nih.gov/35392287/>, <https://pubmed.ncbi.nlm.nih.gov/32029986/>, <https://pubmed.ncbi.nlm.nih.gov/12833112/>). Based on these studies, we developed this binary classification.

3. Even though the manuscript talks about model diagnostics (page 8, lines 150–153), it does not provide variance inflation factors or goodness-of-fit statistics, which are needed to show that the model is good and to check for multicollinearity.

**Response:** Thank you for your comment. As per your suggestion, we have reported multicollinearity and goodness-of-fit statistics for all models considered for three food groups (savory and fried snacks, SFS; sweet snacks, SS; and sugar-sweetened beverages, SSBs), which can be found in supporting Tables S2, S3, and S4. Result section of the manuscript is also updated accordingly (page 13, lines 216-222). The text stated as follows:

***Multicollinearity and goodness-of-fit statistics***

*Multicollinearity statistics for explanatory variables and goodness-of-fit statistics for SFS, SS, and SSBs, separately for adult men and women, are presented in Tables S2, S3, and S4, respectively. The mean VIF values are all less than 10 for all models considered, indicating no multicollinearity among the explanatory variables. Both deviance and Pearson Chi-square statistics showed large p values, indicating there is no lack of fit and suggesting that the Poisson regression models fit the data well.*

4. The analysis includes many comparisons without correcting for multiple tests (page 9, table 4), which raises the chance of getting false positives; think about using a correction method like Bonferroni or FDR.

**Response:** Thank you for your observation. We used 18 predictors in the model and considered p-values <0.05 as statistically significant. Given this, there is less than a 5% chance that one predictor would be significant by chance. Moreover, for many of our factors, the p-value was <0.001. For this reason, we did not apply Bonferroni or FDR-based corrections.

5. The data availability statement confirms full access (page 2, line 35), but the authors are encouraged to enhance transparency by depositing the dataset in a public repository with a DOI.

**Response:** Thank you for your suggestion. Depositing the dataset in [Dryad](http://datadryad.org/) requires a data publishing fee, which we are unable to provide. However, we have shared the dataset as supporting information in both CSV and STATA formats.

6. The manuscript contains grammatical errors, awkward sentence structures, and inconsistent terminology—such as "junk food" versus "unhealthy food"—throughout, including on Page 3, Lines 40–44, and would benefit from professional English editing.

**Response:** Thank you for your observation. However, in this manuscript, we used the term ‘unhealthy food’ to represent (savory and fried snacks, SFS; sweet snacks, SS; and sugar-sweetened beverages, SSBs). We used other terms, such as junk food and fast food, only in the introduction and discussion sections to introduce and explain the broader range of unhealthy foods mentioned by other authors and sources (<https://link.springer.com/article/10.1007/s10597-020-00672-x>, <https://pubmed.ncbi.nlm.nih.gov/38482184/>, <https://pubmed.ncbi.nlm.nih.gov/35816403/>). Regarding English editing we checked and edited the manuscript to correct grammatical errors.

7. The discussion section (pages 17–18) primarily summarizes findings but lacks deeper interpretation through a behavioral or policy lens, particularly regarding gender differences and public health implications.

**Response:** We revised the whole discussion section as per your recommendation. We have added new text also (highlighted in yellow).

8. The limitations section (page 8, lines 154–160) is minimal; it should be expanded to include self-reporting bias, the cross-sectional nature of the study, and lack of data on portion sizes or frequency of consumption.

**Response:** Thank you for your comments on limitation section. We elaborated the section as you suggested in the strengths and limitations section. We added social desirability bias (please see line 428, page 30). We mentioned recall bias (please see line 430, page 30), the cross-sectional nature of the study (line 435, page 30), portion size (line 431, page 30), and the calorie intake issue (line 432, page 30).

9. Tables, especially Table 3 (pages 13–15), are data-heavy; visual representations such as forest plots or bar charts should be added to improve clarity and reader comprehension.

**Response:** Thank you for your comments. As recommended, we have replaced Table 3 with a bar chart (now Fig 2) to present prevalence by gender and area of residence and moved the full table to the supplementary section (S1 Table).
