# Supplementary material for: Unhealthy food consumption among 20–59 years old adults in Bangladesh: Findings from a nationally representative cross-sectional survey
Source: PLoS One. 2025 Dec 2;20(12):e0336984. doi: 10.1371/journal.pone.0336984 (PMC12671833; doi:10.1371/journal.pone.0336984)
Supplement: Attachment — Submitted filename: Response_to_Reviewers_auresp_2.docx [file pone.0336984.s016.docx]

**Manuscript ID: PONE-D-25-00015**

Manuscript title: Unhealthy food consumption among 20-59 years old adults in Bangladesh: findings from a nationally representative cross-sectional survey

**Additional Editor Comments**

1. Thank you for submitting your revised manuscript to PLOS ONE. We appreciate the thoughtful and thorough responses you've provided to both reviewer and editorial comments. The improvements you’ve made—such as adding diagnostic statistics, refining the discussion, and including visual presentations for prevalence estimates—are clear and valuable. Your study tackles an important public health issue using nationally representative data, which makes the findings relevant to a wide audience. That said, there are still a few areas that need further revision before your manuscript can be considered for publication.

**Response:** Thank you for your suggestion. We have revised the manuscript as per your recommendations. Please find the details below.

2. Although you have included CSV and STATA files as supplementary material, we strongly recommend depositing your dataset in a recognized open-access public repository, such as Zenodo or OSF, and obtaining a DOI. This step will promote transparency, increase accessibility, and align your work with best practices in open science. In the methods section, the criteria for defining sufficient versus insufficient physical activity are unclear—the thresholds seem inverted and do not match the WHO guidelines. Please clarify the exact criteria you used and, if they differ from WHO recommendations, provide a clear justification for your approach.

**Response:** As per your suggestion, we have deposited the dataset to OSF, and the DOI is <https://doi.org/10.17605/OSF.IO/YJWNG>

Thank you for your observation. We apologize for this inversion, which occurred due to a typing error in one place in the methods section (Table 1), where instead of “≥150 minutes,” we mistakenly wrote “<150 minutes” as sufficient, while it should have been insufficient. We defined physical activity according to the WHO guideline as follows: Insufficient (<150 minutes of moderate-intensity or <75 minutes of vigorous-intensity physical activity or an equivalent combination of both in a week) and Sufficient (≥150 minutes of moderate-intensity or ≥75 minutes of vigorous-intensity physical activity or an equivalent combination of both in a week) (<https://iris.who.int/server/api/core/bitstreams/d0972fd5-8f7d-4c87-b092-889e0f5f4618/content>).

This threshold has also been used in other studies, including (<https://journals.plos.org/plosone/article?id=10.1371/journal.pone.0251967>). We have corrected this typo in Table 1 (Page 7, line 141) of the methods section.

3. You've included variance inflation factors and model fit statistics in the supplementary tables, which is helpful, but summarizing these results briefly in the main Results section would make the findings more accessible. Including mean VIF values and p-values for deviance and Pearson chi-square directly in the narrative will help readers better understand the robustness of your models. Additionally, the current explanation for not adjusting for multiple comparisons is not sufficient. With so many predictors, the risk of Type I error is real. We suggest either applying a statistical correction, such as Bonferroni or FDR, or providing a more compelling justification for not using these adjustments.

**Response:** We have summarized the mean VIF and the p-values for deviance and Pearson chi-square in the result section (Page 13-14, lines 216–222).

Thank you for your comment. We want to mention that our study is an exploratory study. For an exploratory study, it is not necessary to perform a Bonferroni adjustment (<https://pubmed.ncbi.nlm.nih.gov/11297884/>). Moreover, six predictors with more than two categories are considered in the regression model (age, division, education, wealth quintile, place of residence, and BMI). We compared each category with the reference category and did not make any conclusions based on multiple pairwise tests or confidence intervals.

However, we still obtained Bonferroni-adjusted confidence intervals and p-values, which are provided in the supplementary table (S8, S9, and S10 Table). We did not observe any substantial changes (except for the wealth quintile comparisons between the poorest and richer, and poorest and richest for men; the poorest and poorer, and poorest and middle for women in savory and fried snacks (SFS); the poorest and middle for men, and no formal education compared with primary for women in sweet snacks (SS); and no formal education compared with secondary, poorest compared with highest wealth quintile for men, and poorest compared with middle for women in sugar-sweetened beverages (SSBs)) between the Bonferroni-adjusted p-values and those in the main regression tables; hence, we retained the main table.

4. There are still a few minor grammatical errors, such as “foods intake” instead of “food intake,” and the terminology could be more consistent—consider using “unhealthy food” throughout. A final professional language edit is recommended to help the manuscript read smoothly. While the revised discussion is stronger, it would be even more effective if it were more clearly grounded in behavioral or theoretical frameworks, like nutrition transition or health belief models. Expanding on gender-sensitive interpretations of your results would also add depth. In the policy implications section, try to move beyond general recommendations and suggest specific, practical strategies for Bangladesh, such as taxing sugar-sweetened beverages, introducing front-of-pack labeling, or implementing advertising restrictions. The limitations section is better, but could be further improved by openly acknowledging the likelihood of residual confounding due to unmeasured lifestyle, cultural, or marketing factors.

**Response:** Thank you for your suggestion. We have checked and edited the manuscript to correct grammatical errors. We also revised and enriched the discussion section as per your recommendation and have added new text (highlighted in yellow). We mentioned residual confounding in the limitations section (Page 23, line 432).

5. On presentation, the switch from Table 3 to a bar chart is a great improvement and adds clarity. However, Table 4 is still dense and difficult to interpret. Consider breaking it into smaller, more focused tables, or adding forest plots to make the information clearer for readers. Finally, the funding statement mentions that funders were involved in study design and review. Please clarify exactly how the funders participated and discuss whether their involvement could have introduced bias, so readers have full transparency about this aspect of the research.

**Response:** Thank you for your comment. As you suggested we have replaced table 3,4 and 5 as forest plot (now Fig 3, Fig 4, and Fig 5). However, we moved these tables to the supplementary table (S5 Table, S6 Table, and S7 Table) so that readers can also see the crude prevalence ratio.

Regarding the funders’ involvement, we would like to clarify that they suggested some additional questions for the main questionnaire, attended meetings on data supervision and project updates, and reviewed the manuscript. Their involvement did not affect the study outcomes or interpretation, as all final decisions about the study design, data analysis, and manuscript content were made by the investigators. This ensures transparency and maintains the integrity of the research.

**Reviewer#1 comment**

1. In data availability, please consider depositing the dataset (and code if possible) in a free public repository (e.g., Zenodo, OSF) to obtain a DOI, which will enhance transparency and accessibility.

**Response:** Thank you for your suggestion. We have deposited the dataset to OSF, and the DOI is <https://doi.org/10.17605/OSF.IO/YJWNG>

2. Methods – Physical Activity Definition, kindly clarify the definition and thresholds for sufficient physical activity to ensure alignment with WHO guidelines, or justify the criteria used.

**Response:** We defined physical activity according to the WHO guideline as follows: insufficient (<150 minutes of moderate-intensity or <75 minutes of vigorous-intensity physical activity or an equivalent combination of both in a week) and sufficient (≥150 minutes of moderate-intensity or ≥75 minutes of vigorous-intensity physical activity or an equivalent combination of both in a week) (<https://iris.who.int/server/api/core/bitstreams/d0972fd5-8f7d-4c87-b092-889e0f5f4618/content>).

This threshold has also been used in other studies, including (<https://journals.plos.org/plosone/article?id=10.1371/journal.pone.0251967>). However, due to a typing error in one place in the methods section (Table 1), instead of “≥150 minutes,” we mistakenly wrote “<150 minutes” as sufficient, while it should have been insufficient. We have corrected this typo in Table 1 (Page 7, line 141) of the methods section.

3. Statistical Reporting. Kindly summarize key diagnostic results (mean VIF ranges, p-values for model fit) in the main text for clarity, while retaining full details in the supplementary tables.

**Response:** We have summarized the mean VIF and the p-values for deviance and Pearson chi-square in the result section (Page 13-14, lines 216–222).

4. Language and Terminology, maybe conduct a final proofreading pass to correct minor grammatical issues (e.g., “foods intake” to “food intake”) and ensure consistent use of the term “unhealthy food.”

**Response:** Thank you for your comment. We have checked and edited the manuscript to correct grammatical errors. In this manuscript, we used the term ‘unhealthy food’ to represent (savory and fried snacks, SFS; sweet snacks, SS; and sugar-sweetened beverages, SSBs). We used other terms, such as junk food and fast food, only in the introduction and discussion sections to introduce and explain the broader range of unhealthy foods mentioned by other authors and sources (<https://link.springer.com/article/10.1007/s10597-020-00672-x>, <https://pubmed.ncbi.nlm.nih.gov/38482184/>, <https://pubmed.ncbi.nlm.nih.gov/35816403/>).

5. Discussion and Limitations, kindly add a brief note on potential residual confounding (e.g., unmeasured lifestyle or marketing influences) and acknowledge the bias direction from using binary food classifications.

**Response:** Thank you for your suggestion. We added residual confounding (please see page 23, line 431) and binary food classification (page 23, line 426).
